# Quinquinia—Why It Should Be Used by Country Practitioners

**Published:** 1880-08

**Authors:** James S. Whitmire

**Affiliations:** Metamora, Ill.


### ARTICLE IV.

### QUINQUINIA—WHY IT SHOULD BE USED BY COUNTRY PRACTITIONERS. By JAMES S. WHITMIRE, M.D., Metamora, Ill.

It has always been impressed upon my mind that if there could be prepared from the cinchona bark a preparation containing all the alkaloids of the bark, including all the active principles that remain in the mother waters after the crystalizable alkaloids have been separated—the so-called chinioidine—that we would not only have a better tonic and febrifuge, but that we would establish a system of economy in the use of the bark and its preparations, that is becoming more and more necessary from year to year on account of the destruction of the forests of this tree in their indiginous, or native, haunts. The chief source of the world's supply of the bark has been and still is the South and Central American states. The Colombian states, Peru and Brazil are probably the greatest source of the world's supply of the bark of any of the South American states; and, though the bark is one of the main sources of revenue that some of those states possess, yet the government has taken no steps to prevent the destruction of the trees; besides, it has been ascertained that the bark of the root is more valuable, on account of its being richer in alkaloids than that from other portions of the tree, and, therefore, whole forests of all the species are being swept from the face of the earth on account of this character of vandalism. My son, C. L. Whitmire, who went to Honduras, with others from the Illinois Industrial University, tells me that the cinchona tree grows and flourishes among the mountains and table lands of the isthmus to a considerable extent, but there is so little industry or enterprise among the natives that the bark is not gathered to any great extent, and when it is gathered at all the tree is cut up root and branch, so that in time the total de-destruction of the forests is inevitable. Besides these considerations the instability of the Central and South American governments, their internal feuds, and petty wars with one another all tend to lessen the supply of this now indispensable drug, because one state cannot inflict a greater calamity on another, between which there is a conflict, than to destroy not only the native growth of the cinchona tree, but they not unfrequently destroy the plantations of the tree, where there has been forethought and enterprise enough in the different states to cultivate its growth. Why is it that more of the different governments of the earth have not taken these facts into consideration and begun the cultivation of this invaluable tree? Certainly nearly all must know the growing scarcity of the bark, and also know that without the fostering care of government in its propagation that every species of the tree must become extinct in their native wilds. There is no estimating the value that the bark of the cinchona has been to the world. It has enabled the people to settle in and develop the waste places, and the most valuable of our agricultural lands have been rendered inhabitable by the use of this drug, which, even to this day, would have remained undeveloped. Brazil and Peru are the only two South American states that have made any considerable progress in the cultivation of the cinchona tree.

In this they have shown great wisdom. The British government has shown great forethought and wise counsel in having introduced the cultivation of the tree in her Indian possessions as well as in the island of Jamaica in the West Indies. The Dutch have introduced it, successfully, in the East Indies, and the French in the island of Bourbon and in Algiers. Now that several of the governments have had enterprise and philanthropy enough to banish all doubts as to its successful cultivation, it seems to me incomprehensible why the government of the United States may not follow suit and introduce its culture on her waste lands in the mountainous districts of southern California, Arizona and New Mexico, the climate and other conditions being such as to almost insure its successful culture, it being known that it comes to perfection at an altitude of from 3,000 to 5,000 feet.

During the year 1879 there was more bark imported into the markets of the world than had ever been before, but it was, taken as a whole, vastly inferior in quality, it containing less of the alkaloids than that of former importations. This was owing, partly at least, to the fact that an inferior species of the tree had been cultivated in the Indies, and, also, the vandalism that is permitted in the South American states, in the destruction of the best species of the native tree, for either gain or destruction. The facts as here stated, and which are undeniably true, should induce every physician who prescribes the alkaloids and every manufacturer who places them on the market to economize the products of the cinchona bark, so that no particle of any value whatever should be lost. This has really been the case with the cheaper alkaloids during the last few years, and each has been tried in turn in order to ascertain its true therapeutic value, and now the different alkaloids, exclusive of quinine, are used throughout the Mississippi valley, many physicians, particularly country and village practitioners, using the cheaper alkaloids almost to the entire exclusion of quinine. All the alkaloids, quinine, quinidia, cinchonine and cinchonia, are now in use as tonics and antiperiodics, and for many years the chinoidine has been pressed into the service as a prophylactic, and even as an antiperiodic, which quality it possesses in an eminent degree, though it is not so much used for that purpose, on account of its nauseating qualities, as the other products of the bark. Quinine is now fast becoming, on account of the price, out of the reach of the poorer class of our patients, and had it not been for the cheapness of its congeners, and their very general use, the price of quinine would have been now double what it is. Now, in order to avail ourselves of all the medical constituents of the bark, let us all, particularly country practitioners, who are compelled from necessity, on account of the lack of drug stores, to furnish our own medicines, give the quin-quinia a fair and impartial trial. This preparation of the bark is manufactured by Charles T. White & Co., of New York, and claims to contain all the the alkaloids of the bark, including the amorphous constituents contained in the mother waters after all the crystalizable salts have been separated—the so-called chinoidine. I have been using this preparation for several months in my practice, which extends to and along the Illinois river bottom, consisting of bogs and lagoons. The blood of the people living in the vicinity is, at all seasons of the year saturated with the malaria emanating from the swamps and marshes, so that I have given the quin-quinia a very thorough trial, and, so far, it has never disappointed me in its action. At the commencement of its use I was a little chary in regard to placing entire confidence in its ability to interrupt a paroxysm of ague at an ordinary dose, so that I began at thirty-five grains, divided into seven powders, one to be taken every two, three or four hours, according to the circumstances as to whether it was a quotidian or tertian; then as other cases occurred I continued to lessen the amount used till I found that twenty grains would, in most cases, interrupt a paroxysm of simple intermittent. I soon began to use this preparation in every form of malarial poisoning, complicated or otherwise, when an antiperiodic or a tonic was deemed necessary. Finding that it acted promptly in every instance where it was used, I necessarily became more confident in regard to its action and was prepared to use it under any circumstances and in any emergency.

After I had come to this conclusion it was not long before an opportunity offered to try my faith in the virtues of the new drug. Mr. A. M., aged 30, usually stout, lived on the Illinois river bottom. June 1, 1880, I visited him, immediately, or as soon I could drive eight miles, and found him just rallying from a collapsed condition caused by an attack of pernicious fever—congestive chill. I gave him ten powders consisting each of quin-quinia grs. x., opii. gr. $\frac{1}{5}$, of which one was to be taken every two hours. The next day and the day after my patient missed the paroxysm, and then all the treatment he required was to continue the quin-quinia in from three to five grains every four or five hours during the daytime, so that in a few days he was able to labor about his farm and stable. I have also found that in large doses it has the property of lessening the animal temperature and producing quininism the same, or very nearly so, as that of the sulph. of quinine. Taking it all in all, I am greatly pleased with this new preparation of the bark and its action on the economy; and containing all the active principles of the bark as it is claimed, there is no reason why it may not become the popular remedy, particularly in charity hospitals and with country physicians who furnish their own medicines. It is retailed at one dollar an ounce, and will go two-thirds as far, at least, as an ounce of quinine. Taking its price into consideration it will make quite a difference, annually, in the exchequer of of the country practitioner whose business lies in malarious districts; and so long as public sentiment compels the country practitioner to become the public medical almoner of the poor, it is necessary that we should use every endeavor, in justice to ourselves, where there can be no detriment to the patient, to make our expenses as light as possible, and by the use of this article in appropriate cases, economize the destruction of the bark, and thereby keep quinine and the separate alkaloids at a reasonable figure. While it is true that my experience with the use of quin-quinia has only extended for a few months back, yet it has proven so satisfactory in my hands that I feel it a duty that I owe to my professional brethren to recommend it for trial to all Western practitioners who have to furnish their own antiperodic medicines, believing that it will prove fully, if not more, satisfactory than either of the alkaloids separately, not excepting quinine—the king of antiperodics. Besides, I am particularly impressed in its favor on account of its having all the medical virtues of the bark, as it is claimed, combined in their natural proportions, making less bulk and tasting less bitter than any of the salts of the bark. It is a well known fact that when quinine has failed in chronic malarial poisoning to interrupt the irregular paroxisms, the bark has been resorted to in substance with marked benefit to the sufferer, and has answered the purpose when quinine, the other salts and arsenic combined had failed to effect a cure. These facts, generally known by the profession, are only necessary to be alluded to, to convince every one of the undoubted value of the quinquinia, if it is honestly manufactured and contains all the virtues of the bark as is claimed by its manufacturers.

METAMORA, Ill., June 13, 1880.

---

THE fourth annual meeting of the American Dermatological Association will be held at the Ocean House, Newport, R. I., on the 31st August, the 1st and 2nd September.—ARTHUR VAN HARLINGEN, M.D., Secretary.

